# Doctors and Directors

**Published:** 1891-08-08

**Authors:** 


### The Doctor's Armchair.

### DOCTORS AND DIRECTORS.

Railway directors have never, so far as we know, gone out of their way to show any special favour to doctors. Notwithstanding this fact, one particular doctor has recently conferred very valuable favours on railway directors. Mr. Malcolm McHardy, ophthalmic surgeon to King's College Hospital, read a paper in the Ophthalmological Section of the British Medical Association at Bournemouth last week, in which he dealt at length with the eyesight of railway servants. Professor McHardy has for some time been gathering facts on the subject from the standpoint of his own specialty. He has not merely treated the eyes of railway servants in the hospital wards, but has made himself acquainted with the men and their duties when they have been in health and at work.

By way of illustrating the dangers to which the public are exposed when travelling, Professor McHardy related the story of a signalman who has for years been unable to distinguish a man from a woman at a distance of ten yards. This man had been quite recently re-examined by the railway surgeon, and subjected to the usual tests, with the result that he passed them all successfully, and was put back to his work as signalman, although, so far as the signals were concerned, his eyes were of little more use to him than those of a blind man.

It does not require any overwhelming amount of wisdom to enable a man to understand that Professor McHardy has rendered the public a very important service by bringing this subject forward. But the Professor himself speaks in a kind of half apologetic way, and justifies his action on the grounds that the interests of railway passengers, railway servants, and railway shareholders are all alike profoundly involved in the criminal carelessness which he has brought to light. So far, however, from any apology being needed, the public will feel that it owes Professor McHardy a deep debt of gratitude.

Science, like a new and highly moral religion, makes its way but slowly into the general mind and practice. But there are some scientific discoveries and certainties which demand to be universally applied the very moment they are certainly proved. Such, for instance, was the discovery of chloroform; and such also is the knowledge of antiseptic medicine. The surgeon who should perform a critical and painful operation without giving his patient the option of an anæsthetic would be both cruel and criminal. The physician who, under certain familiar circumstances of septic danger, should neglect to employ antiseptic precautions ought to consider himself a constructive murderer. Similarly, those railway directors, who know that the eyes of men vary much from a normal standard, that certain eyes are absolutely powerless to see and distinguish between particular colours, and that certain others can distinguish nothing at all with any kind of clearness a little distance away, and who know at the same time that science can separate the normal eyes from the abnormal with the utmost precision, and can thus take away at a single stroke one of the chief risks of railway travelling—those directors who know these things and do not use the knowledge which science has placed at their disposal, voluntarily take upon themselves the unnecessary responsibility of smashing their trains, curtailing their shareholders' dividends, and maiming and killing their passengers by the score or by the hundred.

The directors will say, of course, that they employ properly-qualified medical men as railway surgeons, and that those surgeons put the eyes of the signalmen to the usual tests. This plea is as wise as it would be for Lord Wolseley to say that he had taken care to provide his army with the best of bows and arrows, and that some of them had muzzle-loading guns of the most approved eighteenth-century type. The ordinary surgeon, it must be remembered, is for ordinary work; the specialist is for specialism. Nobody would think of taking a man with a colic to an eye doctor; nor, on the other hand, would any experienced person take a man with a cataract to an ordinary parish surgeon. We are not affirming that the specialist is in any way superior to the general practitioner, either as a man, or for all-round practice. He may be, and often is, very inferior. But the specialist, if he be worthy of the name at all, knows his own specialty, and knows it with a completeness and fulness which is not attained by one general practitioner in a thousand. Of course many general practitioners have a considerable amount of special knowledge; and those who are honest will use their knowledge and experience so far as it will serve them; and when they have come to the end of it they will frankly tell their patients so, and advise the calling in of a specialist.

The point for railway directors and all other persons to bear in mind is this, that where dangerous risks are involved it is imperative that the highest obtainable knowledge and skill shall be employed, with the view of reducing those risks to a minimum. This proposition surely needs no arguing! In the case under consideration, of railway signal men, there is no difficulty whatever in guarding against the dangers involved in employing men of defective vision. There are competent oculists in every considerable town. The certificate of one or more such oculists should be compulsory for all those who seek employment in any position where perfect eyesight is imperatively demanded. Of course, one would naturally think that these things would be as obvious to railway directors as to us, and that the last thing that would be required would be an exhortation to the performance of such manifest duty. But the facts are all the other way. Railway directors, like other men, are indolent, obstinate, dull-witted, and selfish. They require to have spectacles furnished to enable them to see their own interests, and whips and goads to drive them forward in the path of their own safety and prosperity. Shareholders are generally more stupid, blind, and greedy, than even directors. The public, however, has interests which it must care for, whatever becomes of directors' fees and shareholders' dividends. Its great interest is the preservation of its limbs and life. The press, as the guardian of the public interests, should take care to report and signalise every railway accident, great and small, which can be traced to the defective eyesight of a signalman. To the energetic and constant action of the press, both medical and lay, we must look for the speedy carrying into effect of these immediately urgent measures of public safety.

